# MRI-Based Morphological Features as Predictors of Clinical Outcomes in Locally Advanced Rectal Cancer Treated with Neoadjuvant Chemoradiotherapy: Insights from a Single-Institution Experience

**DOI:** 10.3390/jcm15020430

**Published:** 2026-01-06

**Authors:** Marco Lucarelli, Consuelo Rosa, Giulia de Pasquale, Monica Di Tommaso, Tamara Santone, Antonietta Augurio, Angelo Di Pilla, Marianna Nuzzo, Maria Taraborrelli, Marianna Trignani, Annamaria Vinciguerra, Andrea Delli Pizzi, Marta Di Nicola, Domenico Genovesi, Andrea D’Aviero

**Affiliations:** 1 Division of Radiation Oncology, European Institute of Oncology IRCSS, 20141 Milan, Italy; 2 Department of Radiation Oncology, “S.S. Annunziata” Chieti Hospital, 66100 Chieti, Italy; consuelo.rosa@asl2abruzzo.it (C.R.); giuliadepasquale@hotmail.it (G.d.P.); monica.ditommaso@asl2abruzzo.it (M.D.T.); tamara.santone@graduate.univaq.it (T.S.); antonietta.augurio@asl2abruzzo.it (A.A.); angelo.dipilla@asl2abruzzo.it (A.D.P.); marianna.nuzzo@asl2abruzzo.it (M.N.); taraborrellimaria@gmail.com (M.T.); marianna.trignani@asl2abruzzo.it (M.T.); annamaria.vinciguerra@asl2abruzzo.it (A.V.); d.genovesi@unich.it (D.G.); andrea.daviero@unich.it (A.D.); 3 Department of Innovative Technologies in Medicine and Dentistry, “G. d’Annunzio” University, 66100 Chieti, Italy; andrea.dellipizzi@unich.it; 4 ITAB Institute for Advanced Biomedical Technologies, “G. d’Annunzio” University, 66100 Chieti, Italy; 5 Department of Medical, Oral and Biotechnological Sciences, “G. d’Annunzio” University of Chieti, 66100 Chieti, Italy; marta.dinicola@unich.it

**Keywords:** rectal cancer, magnetic resonance imaging, outcomes prediction

## Abstract

**Objectives:** This study evaluates MRI-based morphological features as predictors of long-term clinical outcomes in patients with locally advanced rectal cancer (LARC) treated with neoadjuvant chemoradiotherapy (CRT). **Methods:** A retrospective analysis was performed on 134 patients treated between 2014 and 2024. Patients underwent dose-intensified radiotherapy (55 Gy) with concurrent capecitabine followed by surgery. Radiological features analyzed on pre- and post-CRT MRI included Tumor Extension Beyond Muscularis Propria (TEMP), Circumferential Resection Margin (CRM), Extramural Venous Invasion (EMVI), and Lateral Lymph Nodes (LLN). **Results:** Five-year Overall Survival (OS), Disease-Free Survival (DFS), and Local Control (LC) rates were 85%, 83%, and 88%, respectively. Patients with TEMP > 5 mm had significantly worse LC (*p* = 0.02) and DFS (*p* = 0.04). A positive CRM (<1 mm) significantly correlated with reduced DFS (*p* = 0.04). The presence of EMVI was associated with significantly lower LC (*p* = 0.01). Additionally, persistent pathological LLN after treatment significantly impacted LC (*p* = 0.04). **Conclusions:** MRI morphological features such as TEMP > 5 mm, CRM < 1 mm, EMVI, and pathological LLN are significant predictors of worse oncological outcomes. Identifying these imaging biomarkers allows for better risk stratification and personalized treatment strategies in LARC.

## 1. Introduction

### 1.1. Background

Globally, colorectal cancer ranks as the third most frequently diagnosed malignancy and represents the second most common cause of cancer mortality [1]. Throughout Europe in 2020, this disease comprised 12.7% of newly diagnosed cancers and accounted for 12.4% of cancer-related deaths [2]. Within Italy, it represented 11.6% of all new cancer diagnoses and 10.8% of all cancer deaths during the same timeframe [3].

For patients with locally advanced rectal cancer (LARC), defined as stage II (tumor invading the muscular wall without lymph node involvement) and III (with lymph node involvement), specifically cT3 N0 and cT3-4 N0-2 M0 tumors, the established treatment approach involves long-course preoperative chemoradiotherapy (CRT) followed by surgical resection. The landmark German CAO/ARO/AIO-94 phase III randomized trial demonstrated that a preoperative approach achieves high local control (LC) rates (5-year LC of 94%), higher sphincter preservation rates (39% vs. 19%), and lower incidence of both acute (27% vs. 40%, *p* = 0.001) and late toxicity (14% vs. 24%) compared to postoperative treatment [4]. However, no difference was observed between the two treatment schedules regarding disease-free survival (DFS) (36% vs. 38%) and overall survival (OS) (76% vs. 74%) [4].

Artificial intelligence (AI), encompassing machine learning (ML), artificial neural networks (ANNs), and deep learning (DL), has emerged as a transformative force in medical imaging and oncology [5]. ML involves training algorithms on data to make predictions without explicit programming, while DL uses multi-layered neural networks to process complex imaging datasets [5]. In rectal cancer imaging, AI applications span two complementary approaches: (1) traditional radiological assessment of morphological features based on established guidelines, and (2) computational radiomics involving automated extraction of quantitative imaging features through ML/DL algorithms.

True radiomics, as distinct from conventional morphological assessment, involves high-throughput extraction of hundreds of quantitative features from medical images—including texture patterns, shape descriptors, intensity distributions, and higher-order statistical features—that may not be visually perceptible to human observers [6,7,8,9,10,11]. These features are then analyzed using ML algorithms to develop predictive models. Recent DL approaches utilizing convolutional neural networks (CNNs), such as ResNet-101 and Vision Transformers (ViT), have demonstrated superior performance in predicting lymph node metastasis and other oncological outcomes in rectal cancer, achieving areas under the curve (AUC) ranging from 0.819 to 0.961, significantly outperforming conventional radiologist assessments [6,8]. These AI-driven approaches enhance model interpretability through tools such as Shapley Additive exPlanations (SHAP), allowing clinicians to understand the decision-making process underlying predictions [9]. Studies have demonstrated that radiomic models combining multiparametric MRI features can predict distant metastasis with C-indices up to 0.775 [8] and enhance the predictive accuracy of OS from 0.672 using only clinical features to 0.730 when incorporating radiomic features [11].

However, clinical implementation of AI-based radiomics faces several challenges, including: (1) the need for large, standardized training datasets, (2) computational expertise and infrastructure requirements, (3) limited interpretability of black-box models, and (4) validation across different institutions and MRI protocols [5]. While tools such as SHAP enhance model interpretability [9], the clinical integration of fully automated radiomic models remains an evolving field.

In parallel, conventional morphological MRI features—such as those evaluated in our study—represent established, guideline-supported imaging biomarkers that are routinely assessed in clinical practice [12,13]. These features, while based on visual assessment rather than computational extraction, provide clinically validated prognostic information and inform treatment decisions in real-world settings. The European Society of Gastrointestinal and Abdominal Radiology (ESGAR) and other international guidelines mandate reporting of these morphological characteristics for rectal cancer staging [13].

Despite the proven efficacy of neoadjuvant treatment demonstrated since 2012 [4], clinical practice reveals that patients with the same clinical stage treated with the same therapeutic protocol have different outcomes. This heterogeneity in treatment response highlights the need for better predictive biomarkers, whether derived from conventional morphological assessment or emerging AI-based techniques.

### 1.2. Tumor Extension Beyond Muscularis Propria (TEMP)

Tumors receive T3 classification when breach of the muscularis propria occurs with tumor extension beyond the rectal wall into the mesorectal fat. T3 lesions undergo further subdivision into four categories determined by the distance from the muscularis propria to maximum extramural invasion: T3a < 1 mm, T3b 1–5 mm, T3c 5–15 mm, and T3d > 15 mm. However, tumors spreading ≤ 5 mm into the mesorectum have local recurrence and survival rates similar to those limited to the muscularis propria (T2) [12]. Indeed, as documented by Siddiqui and colleagues in their meta-analysis, patients with extension beyond the muscularis propria > 5 mm showed lower rates of OS and DFS and LC [14].

### 1.3. Circumferential Resection Margin (CRM)

Among the most critical imaging markers for predicting local recurrence and serving as a reliable prognostic factor for DFS and OS is the circumferential resection margin (CRM), characterized as a distance of 1 mm or less from the tumor or irregular lymph nodes to the mesorectal fascia, as established by the European Society of Gastrointestinal and Abdominal Radiology guidelines [13] and the North American Society of Abdominal Radiology [15]. This marker plays such an important role because the outcome of the standard total mesorectal excision (TME) surgery depends on the relationship of the tumor to the mesorectal fascia. The MERCURY study, a prospective multicenter study, analyzed 374 patients and observed that the LC rate in CRM-positive patients was 80%, compared to 93% in CRM-negative patients, and 5-year OS was 62.2% in CRM-negative patients compared to 42.2% in CRM-positive patients [16].

### 1.4. Extramural Venous Invasion (EMVI)

An additional imaging marker with substantial predictive value for treatment response and clinical outcomes is extramural venous invasion (EMVI). This marker demonstrates strong correlation with disease recurrence rate and, specifically, with both synchronous and metachronous systemic disease dissemination [17,18]. Positive EMVI is correlated with reduced DFS, with stage II EMVI-positive disease showing similar results to stage III EMVI-negative disease [19]. Furthermore, a recent study suggests that EMVI is correlated with lymph node metastases: multivariate analyses demonstrated a higher risk of lymph node metastases in EMVI-positive patients (*p* = 0.0112) [20]. Such robust predictive validity has led ESGAR to recommend defining the presence of EMVI both before and after neoadjuvant treatment [13]. This approach enables preoperative stratification of patients into low- or high-risk categories and facilitates a more personalized treatment strategy for a significant proportion of the rectal cancer patient population, with a mean EMVI prevalence of 26%, as reported in the systematic review of Chand and colleagues, 2016 [21].

### 1.5. Lateral Lymph Nodes (LLN)

Rectal cancers situated below the peritoneal reflection demonstrate a propensity to spread laterally to lymph nodes surrounding the internal iliac and obturator vessels [22]. These lateral lymph nodes (LLN) are located outside the standard surgical plane of total mesorectal excision (TME). In the era before neoadjuvant CRT and TME, local recurrences (LR) occurred frequently and were often localized in the pelvis [23]. However, nowadays, although pelvic LC shows very high rates, exceeding 90%, the absolute risk of LR is approximately 50% in the lateral compartments [8,11]. This is most likely due to LLN not yet being appropriately treated. In 30–40% of patients with primarily enlarged LLN (>10 mm, short axis) treated with CRT and TME, LR occurs within 5 years [8]. A recent international cohort of 1216 patients with standardized review of all MRIs found that patients with enlarged LLN (≥7 mm) before CRT had a 5-year LR rate of 19.5% [6]. LLN ≥ 7 mm before CRT in the internal iliac compartment that remained >4 mm at restaging had a 5-year LLR rate of 52.3%. Obturator LLN had a 5-year LLR risk of 17.8% when remaining > 6 mm. Only 22% of internal iliac LLN significantly reduced in size (<4 mm) at restaging, compared to 63% of obturator LLN [24,25]. Recent radiomic studies have shown promising results in predicting lateral pelvic lymph node metastasis, with MRI-based radiomic models achieving AUCs of 0.819–0.821, significantly outperforming conventional radiologist assessments [6].

### 1.6. Aims

Our study’s objective was to retrospectively examine data from patients who received treatment during the past decade with dose-intensified radiotherapy (RT) and concomitant capecitabine in a neoadjuvant long-course CRT program. We assessed long-term oncological outcomes including OS, DFS, and LC. Subsequently, we examined conventional morphological characteristics of pre- and post-CRT MRI as predictive factors for clinical outcomes and response. Our investigation concentrates on guideline-based morphological features that can be evaluated through visual interpretation by trained radiologists, providing immediately applicable prognostic information.

## 2. Materials and Methods

### 2.1. Patient Selection

Between 2014 and 2024, we retrospectively examined 134 patients with LARC who were admitted to the Radiation Oncology Department of Chieti and underwent preoperative CRT followed by surgery. All enrolled patients were >18 years old, possessed histologically confirmed primary rectal adenocarcinoma and no extrapelvic disease (TNM staging as cT2-4 cN0-2). We reviewed all available medical records in both digital and paper format for data collection.

Pre- and post-CRT staging utilized physical examination, digital rectal examination, and chest-abdomen-pelvis CT scan. Throughout the 5 years following CRT, rectal MRI was performed routinely.

### 2.2. Radiotherapy and Concomitant Chemotherapy

RT was administered using the volumetric modulated arc therapy (VMAT) technique, delivering a total dose of 45 Gy, 1.8 Gy/day, to the pelvic lymph nodes and 55 Gy, 2.2 Gy/day to the entire mesorectum in simultaneous integrated boost (SIB), corresponding to an equivalent dose at 2 Gy/fraction (EQD2) of 57.5 Gy (considering α/β = 5.06 Gy for rectal tumor). During simulation, patients were immobilized supine and instructed to consume 750 mL of water over 45 min to achieve appropriate bladder volume. The clinical target volume (CTV) encompassed the primary tumor and mesorectal, presacral, and pelvic lymph nodes up to the L5/S1 junction. The CTV Boost was delineated to include the primary tumor and mesorectum. The planning target volume (PTV) and PTV Boost comprised their corresponding CTV plus an 8 mm margin in all directions. Dose specification followed the International Commission on Radiation Units and Measurements (ICRU 50–62) report. All patients received concomitant chemotherapy: Capecitabine 825 mg/m^2^, twice daily for 5 days/week during radiation treatment.

### 2.3. Surgery

Radical surgery, including anterior resection (AR) with total mesorectal excision (TME) or abdominoperineal resection (APR), with colorectal or colo-anal anastomosis, was performed according to surgical assessment.

### 2.4. Adjuvant Chemotherapy

Adjuvant chemotherapy was administered to 47 patients (26%). Specifically, 39 patients received capecitabine and 4 capecitabine + oxaliplatin, while FOLFIRI + bevacizumab, FOLFIRI + panitumumab, FOLFOX regimens, and gemcitabine were administered to 1 patient each, respectively.

### 2.5. Toxicity

Radiation Therapy Oncology Group (RTOG) toxicity criteria were used to evaluate acute RT toxicities. Routine postoperative follow-up examinations were performed every 6 months during the first 5 years following surgery, then annually. Gastrointestinal, urinary, hematological, and cutaneous symptoms were evaluated at baseline, during treatment, and at each follow-up examination. Late toxicities were reported according to the RTOG/European Organization for Research and Treatment of Cancer (EORTC) scoring system [26].

### 2.6. MRI Radiomic Features

Patients underwent MRI at two time points: before CRT initiation and between 8–10 weeks after treatment completion, defined as pre-CRT MRI and post-CRT MRI, respectively.

A multidisciplinary team consisting of an experienced radiologist, junior radiologists, an experienced radiation oncologist, and a junior radiation oncologist performed consensus review of all available MRIs from the study population. Measurements and assessments were performed collaboratively using a consensus approach, with preliminary evaluations by junior team members followed by review and final determinations made by the senior radiologist and radiation oncologist when disagreements arose. All assessments were based on established international guidelines [12,13].

The following morphological characteristics were recorded: tumor extension beyond muscularis propria (TEMP), circumferential resection margin (CRM), extramural venous invasion (EMVI), and lateral lymph nodes (LLN).

According to national and international guidelines [12,13,27], we divided patients into: TEMP < 5 mm and >5 mm. Also for CRM, since it is defined as positive if there is a distance of 1 mm or less between the pathological lesion and the mesorectal fascia [12,13], patients were divided into: CRM < 1 mm and CRM > 1 mm. For LLN, the indications for pathological lymph node cut-off provided by Ogura et al. [28] and Sluckin et al. [29] were followed: LLN > 7 mm in the pre-CRT phase and LLN > 4 mm in the post-CRT phase were considered pathological. Finally, patients with pre-CRT LLN > 7 mm and post-CRT LLN < 4 mm were classified as responders, and patients with pre-CRT LLN > 7 mm and post-CRT LLN > 4 mm as non-responders.

### 2.7. Statistical Analysis

Descriptive statistics were outlined as frequencies and percentages, while all numerical variables were reported as means and standard deviation (SD). Survival analyses for 5- and 10-year OS, DFS, and LC rates were performed using the Mantel-Cox test. OS was defined as the time interval between surgery and death; DFS was defined as the time between surgery and the first event (recurrence and/or distant metastasis), and LC was considered as the time between surgery and locoregional recurrence. For patients in whom none of the events occurred, the observation time interval was defined as the period from surgery to the last follow-up visit. The Mantel–Cox test was also used to estimate 5- and 10-year OS, DFS, and LC after stratifying patients by MRI characteristic subgroups. A *p* < 0.05 was considered statistically significant.

### 2.8. AI Statement

During the preparation of this manuscript, the authors used Claude Sonnet 4.5 for the purposes of improvement of language and of readability. The authors have reviewed and edited the output and take full responsibility for the content of this publication.

## 3. Results

### 3.1. Patients’ Characteristics (Table 1)

A total of 134 patients were analyzed in this study. Mean patient age was 67.5 years (range: 37–94 years); 86 patients (64%) were male with a male/female ratio of 1.8:1. Mean Karnofsky Performance Score (KPS) was 96% (range: 70–100%).

**Table 1:** Patients’ characteristics.

		Value (n)
Age, year old	Median, range	67 (37–94)
Gender	Male	86
	Female	47
KPS, %	Median	96
Clinical stage T	T2	9
	T3	113
	T4	12
Clinical stage N	N0	21
	N1	45
	N2	68
Grade	G1	16
	G2	69
	G3	10
	Not available	35
Surgery	AR	92
	APR	26
	Others	6
	Not available	10
Pathological stage T	T0	45
	T1	11
	T2	28
	T3	37
Pathological stage N	N0	80
	N1	18
	N2	4
TRG	1	46
	2	36
	3	22
	4	16
	5	1

Abbreviations: KPS: Karnofky performance score; AR: Anterior resection; APR: Abdominoperineal resection; TRG: Tumor regression grade.

Rectal bleeding was the most common presenting symptom in the study population (72%), followed by pelvic pain and/or tenesmus (26%). Most patients (82%) had cT3 tumors and, simultaneously, 51% of patients presented with cN2 lymph node stage. The most frequent histological presentation was adenocarcinoma (90%), while the most represented histological grading was G2 (53%).

### 3.2. Radiotherapy and Concomitant Chemotherapy

All patients underwent radiation treatment with dose intensification up to 55 Gy, associated with capecitabine. Treatment was interrupted in 10 patients due to toxicity. Mean follow-up was 45 months (range 4–102).

### 3.3. Surgery and Adjuvant Chemotherapy

One hundred twenty-four patients (92%) underwent surgery. Anterior resection was performed in 92 patients (74%), while 26 patients (20%) underwent abdominoperineal resection. Three patients (2.2%) with favorable clinical stage (cT2N0) and a major pathological response chose a watch-and-wait approach. Other types of surgery were performed in five patients (3.7%), while eight patients (6%) did not undergo surgery due to poor clinical conditions. Adjuvant chemotherapy was administered to 34 patients (26%).

### 3.4. Acute Toxicity

The most frequent acute toxicity reported was lower gastrointestinal: 28 patients (21%) with grade 2 toxicity, while toxicity grade ≥ G3, such as rectal bleeding and/or severe diarrhea, was not reported in any patient. The second most encountered acute toxicity was genitourinary with a total of six patients with grade 2 toxicity. Following this, acute skin toxicity was reported in three patients experiencing grade 2, while grade G3 toxicity (moist desquamation) was reported in two patients. No severe hematological, neurological, or hepatic toxicities occurred (Table 2).

### 3.5. Late Toxicity

According to the RTOG/EORTC scale, five patients (4%) reported severe late intestinal toxicity, with bleeding requiring surgical treatment. Moderate diarrhea (more than 5 episodes per day) occurred in one patient (0.7%). Moderate pollakiuria (1 urination < 2 h) and/or intermittent macroscopic hematuria was reported in one patient (0.7%). No other severe late toxicities were reported (Table 3).

### 3.6. MRI Morphological Features

From the review of magnetic resonance imaging by the group of radiologists and radiation oncologists, Table 4 was obtained.

Of 134 patients enrolled, MRI data was unavailable for review in 41 patients (31%). Specifically, 12 patients (9%) did not undergo MRI due to contraindications: pacemaker carrier (n = 4), metallic prostheses (n = 3), severe claustrophobia (n = 3), or patient refusal (n = 2). Twenty-nine patients (22%) had MRI performed but images were not available for retrospective review due to archival system limitations or incomplete digital transfer, particularly for examinations performed in the earlier years of the study.

Given the missing-at-random pattern, we performed complete-case analysis for each MRI morphological characteristic. The specific sample sizes for each analysis are: Pre-CRT TEMP/CRM measurements: n = 93 (69% of total cohort); Post-CRT TEMP/CRM measurements: n = 101 (75% of total cohort); Pre-CRT EMVI assessment: n = 102 (75% of total cohort); Post-CRT EMVI assessment: n = 102 (75% of total cohort); Pre-CRT LLN assessment: n = 25 (19% of total cohort), and Post-CRT LLN assessment: n = 11 (8% of total cohort).

From the analyses, patients who showed TEMP > 5 mm were 51 pre-CRT and 20 post-CRT. A CRM < 1 mm was observed in 44 patients pre-CRT and 23 post-CRT. EMVI was recorded in 62 patients before CRT initiation and in 38 patients at CRT completion. Regarding LLN, 25 patients presented pathological lateral compartment lymph nodes > 7 mm (range: 8–18 mm). Of these, 14 had regression of lymph node metastases, while in 11 persistence of lateral lymph node pathological status with lymph nodes > 4 mm (range: 5–16 mm) was observed.

## 4. Outcomes

Five-year OS, DFS, and LC rates were 85%, 83%, and 88%, respectively. Long-term results at 10 years showed OS, DFS, and LC rates of 75%, 81%, and 88%, respectively.

### 4.1. Impact of TEMP on Outcomes

**Figure 1:**
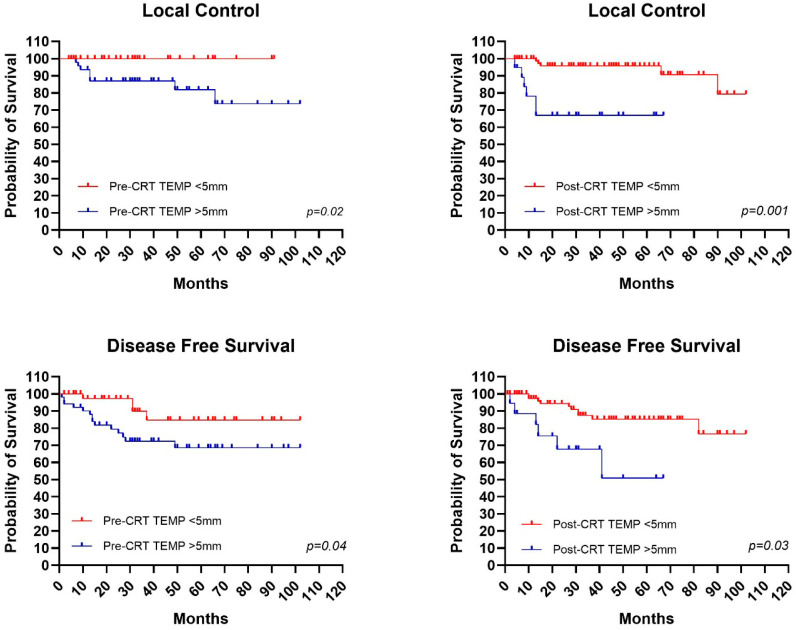
Mantel–Cox test for statistical significance in patients with and without tumor extension beyond the muscularis propria (TEMP) for pre- and post-chemoradiotherapy local control curves, and for pre- and post-chemoradiotherapy disease-free survival curves.

Regarding pre-CRT MRI radiological characteristics, survival outcome analyses were conducted. Starting with TEMP, the subgroup with TEMP < 5 mm presented 5-year LC, DFS, and OS of 100%, 85%, and 82% versus values of 82%, 68%, and 66% for the subgroup with TEMP > 5 mm (Figure 1). Ten-year outcomes were as follows: for patients with TEMP < 5 mm: LC: 100%, DFS: 85%, and OS: 58%, while for patients with TEMP > 5 mm, LC: 73%, DFS: 68%, and OS: 55%. Statistical significance was found in LC curves with *p* = 0.02 and DFS with *p* = 0.04. From analyses conducted on post-CRT MRI data, it was found that in the group with TEMP > 5 mm, 5-year LC and DFS values of 65% and 52% were obtained, compared to the group with TEMP < 5 mm, which presented 5-year LC and DFS values of 96% and 85%, with statistical significance of *p* = 0.001 and *p* = 0.03, respectively, for LC and DFS curves (Figure 1).

### 4.2. Impact of CRM on Outcomes

**Figure 2:**
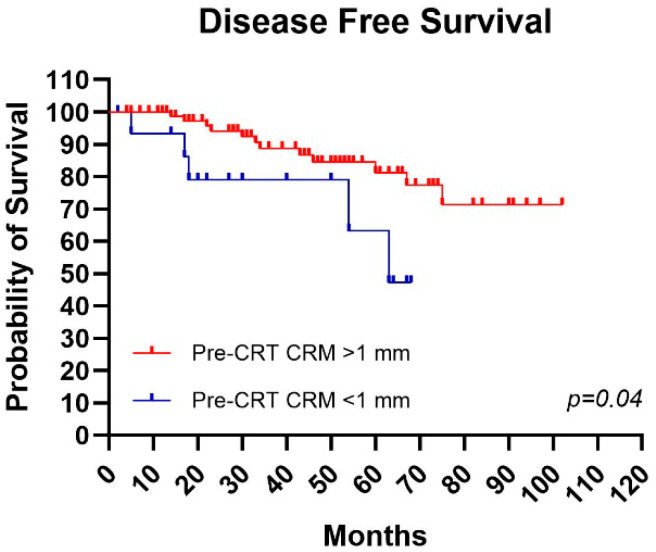
Mantel–Cox test in patients with and without circumferential resection margin (CRM) involvement: *p*-value = 0.04.

Speaking of CRM, in patients with CRM > 1 mm, 5-year LC, DFS, and OS rates of 91%, 80%, and 85% were recorded, as well as 10-year LC, DFS, and OS rates of 91%, 79%, and 60%. Instead, in patients with CRM < 1 mm, recorded LC rates were 89% at 5 years and 54% at 10 years; DFS 74% at 5 years and 74% at 10 years; and finally, OS 67% at 5 years and 53% at 10 years (Figure 2). Statistical significance was obtained in the curves for DFS (*p* = 0.04).

### 4.3. Impact of EMVI on Outcomes

**Figure 3:**
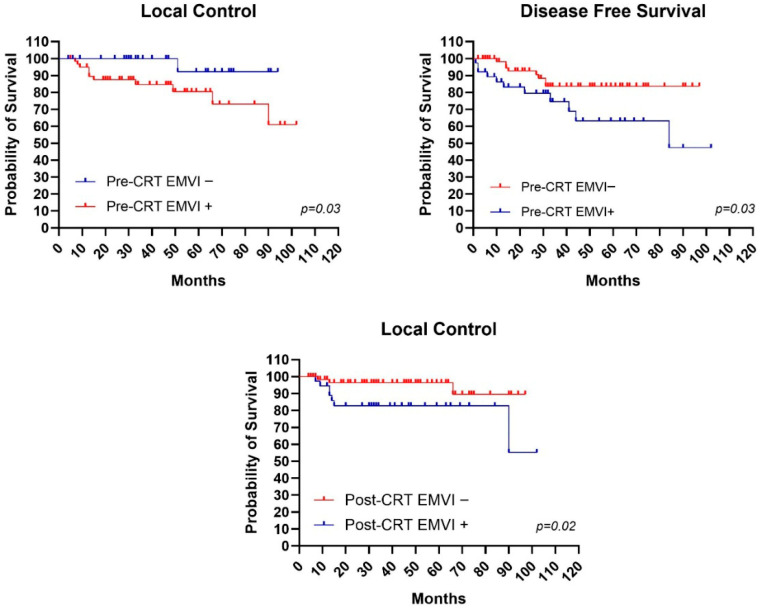
Mantel–Cox test with statistical significance in patients with and without extramural vascular invasion (EMVI) for local control curves pre- and post-chemoradiotherapy and for disease-free survival curves pre-chemoradiotherapy.

Regarding EMVI, patients who did not present vascular invasion showed LC, DFS, and OS values of 100%, 78%, and 84% at 5 years and values of 100%, 78%, and 60% at 10 years. In comparison, patients with presence of vascular invasion showed LC, DFS, and OS values of 80%, 73%, and 64% at 5 years and values of 61%, 73%, and 52% at 10 years. Statistical significance was found in LC curves with *p* = 0.01 (Figure 3).

From analyses conducted on post-CRT MRI data, it was observed that 5-year LC and DFS rates were 83% and 52% for patients with presence of EMVI versus 96% and 67% for patients without presence of EMVI (Figure 3).

### 4.4. Impact of Lateral Lymph Nodes on Outcomes

**Figure 4:**
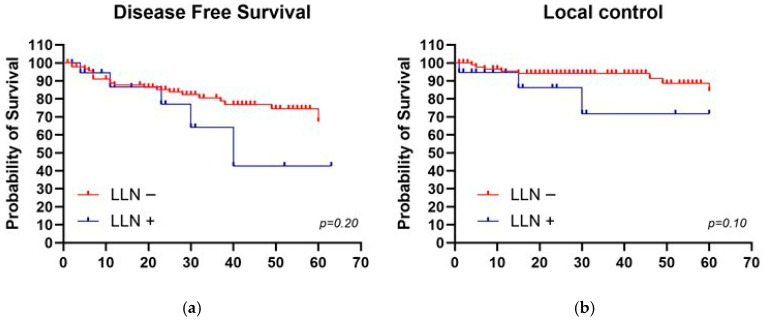
Mantel–Cox test in patients with and without pathological lateral compartment lymph nodes (LLN > 7 mm) for (**a**) Disease-free survival; (**b**) Local control.

Regarding lateral compartment lymph node involvement, the analysis conducted between LLN+ pre-CRT vs. LLN− pre-CRT groups showed promising results for LC and DFS with *p* values = 0.10 and 0.20, respectively. Five-year LC and DFS results were, for LLN+, 77% and 43% versus 87% and 78% for LLN−, respectively (Figure 4).

**Figure 5:**
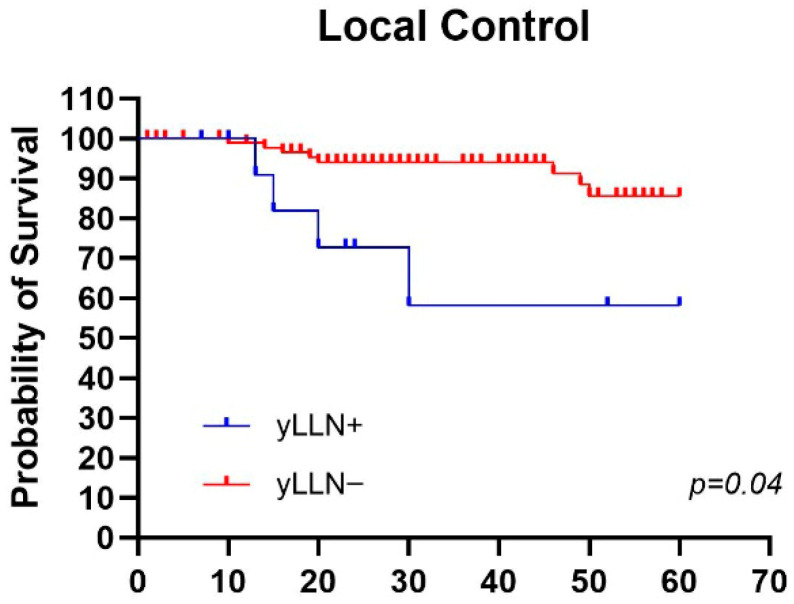
Mantel–Cox test in patients with and without pathological lateral compartment lymph nodes after neoadjuvant treatment (LLN > 4 mm) for Local control.

Regarding post-treatment analysis, in the group of patients with persistence of LLN+, we recorded a statistically significant difference in LC with *p* value = 0.04. Five-year LC results were, for post-CRT LLN+, 58% versus 87% for post-CRT LLN− (Figure 5). No statistical difference was found for post-treatment DFS.

## 5. Discussion

In LARC, neoadjuvant radio-chemotherapy has the potential to determine high LC with a good probability of obtaining better quality and life expectancy [30,31]. Yet, although neoadjuvant treatment has demonstrated its important efficacy since 2012, clinical practice shows that patients with the same clinical stage and treated with the same therapeutic protocol have different outcomes. From the desire to understand which factors can influence treatment response and outcomes, this study was born with the aim of describing our Center’s experience in treating RC and analyzing radiological factors in our study population that in the literature have been shown to have predictive value.

The integration of MRI-based radiomic features in clinical practice represents a significant advancement in personalized medicine for RC patients. Recent literature supports the use of multiparametric MRI radiomics for outcome prediction, with studies demonstrating that radiomic models can enhance predictive accuracy beyond traditional clinical and imaging parameters [11,24,25,32,33].

### 5.1. Tumor Extension Beyond Muscularis Propria (TEMP)

The extent of mesorectal tumor invasion, in other words, TEMP, has been demonstrated as an independent risk factor in numerous studies [16,34,35]. The meta-analysis conducted by Siddiqui et al. in 2018 observed that tumors with invasion greater than 5 mm from the muscularis propria had statistically significantly worse OS and statistically worse DFS [14]. At the same time, in patients with less invasion beyond the muscularis propria, higher OS was observed (*p* < 0.01) [14]. This data appears to be confirmed by Katsumata et al.’s study where statistical significance was recorded for both 5-year DFS and OS, reporting in TEMP > 5 mm patients DFS and OS values of 60% and 53% versus values of 85% and 93% in patients with TEMP < 5 mm [36]. The correlation of tumor invasion into adipose tissue with clinical outcomes, particularly with DFS, was also observed in Shin et al.’s study where a statistically significant difference was reported between patients with TEMP > 5 mm and those with TEMP < 5 mm (5-year DFS: 77.6% vs. 55.2%, *p* < 0.001) [37]. Merkel et al. also observed a similar difference in their work, with 5-year LC, OS, and DFS rates in T3a patients (TEMP < 5 mm) being 93%, 79%, and 85% compared to values of 82%, 74%, and 54% in T3b patients (TEMP > 5 mm) [34].

In our experience, we observed a difference between the TEMP < 5 mm subgroup with 5-year LC, DFS, and OS of 100%, 85%, and 82% versus the TEMP > 5 mm subgroup with values of 82%, 68%, and 66%. Furthermore, statistical significance was detected for LC curves (*p* = 0.02) and DFS (*p* = 0.04). These findings align with recent radiomic studies showing that quantitative assessment of tumor extension provides valuable prognostic information beyond conventional T-staging [38].

### 5.2. Circumferential Resection Margin (CRM)

CRM has been defined as the most important among radiological features regarding LC and as a predictive factor for systemic disease spread. The MERCURY trial was the first study to analyze the reliability of MRI methodology in describing this feature and to study its predictive value. The results were OS and DFS for all patients with CRM < 1 mm of 68% and 85%, respectively [16]. The LC rate for this group of predicted patients was 97% [16]. From these bases, Patel et al. conducted their study observing a 5-year LC rate of 88% in CRM > 1 mm patients and 72% in those with CRM < 1 mm [39]. A difference was also observed in this patient group for OS and DFS with rates at mean follow-up time (50 months) of 59% and 58% for negative CRM and 46% and 51% for positive CRM [39]. The review conducted by Wolberick even states that knowing preoperative CRM status is more important than T-stage classification for its predictive value and for the help it provides to the multidisciplinary group in therapeutic decision-making. In that review, the CRM-positive group presented 5-year OS, DFS, and LC rates of 40–70%, 62–58%, and 61–91% compared to 5-year rates in the CRM-negative group of OS, DFS, and LC of 74–90%, 88–94%, and 90–100% [40].

In our study, in patients with CRM > 1 mm or negative CRM, LC, DFS, and OS rates of 91%, 80%, and 85% at 5 years were recorded. Instead, in patients with CRM < 1 mm or positive CRM, 5-year recorded LC rates were 89%; 5-year DFS 74%; and finally, 5-year OS 67%. Despite differences between subgroups and values comparable to the literature, curves with statistical significance were not obtained for OS and LC, though DFS showed statistical significance (*p* = 0.04).

### 5.3. Extramural Venous Invasion (EMVI)

Another MRI characteristic that strongly correlates with both LC and systemic disease spread is EMVI. Indeed, as demonstrated by Chand et al., positive EMVI is correlated with reduced DFS (3-year rate: 59% compared to 79% in EMVI-negative patients), and it was also observed that stage II but EMVI-positive disease shows similar results to stage III and EMVI-negative disease [19]. In their study, Patel et al., among the evaluated variables, observed how EMVI status appears to be the most important factor predicting both DFS and recurrence incidence. Three-year DFS for EMVI-positive patients was 44% versus 96% for EMVI-negative patients (*p* = 0.0001) [41]. Three-year cumulative recurrence incidence for EMVI-positive patients was 44% versus 4% for EMVI-negative patients (*p* = 0.0019) [41]. This impact on DFS is also present in Sun et al.’s study where DFS was significantly lower in EMVI-positive patients compared to EMVI-negative patients (3-year DFS: 58% versus 90% *p* = 0.013) [42]. However, OS was not significantly lower in EMVI-positive patients compared to EMVI-negative patients (*p* = 0.420) [42]. Finally, Kim et al.’s study also demonstrates the strong predictive value of EMVI on DFS with 3-year rates of 89% in EMVI-negative patients and 55% in EMVI-positive patients [43].

Recent advances in automated deep learning pipelines for EMVI classification on baseline MRI have shown promise in multi-center studies, potentially improving standardization and reproducibility of EMVI assessment [44].

Regarding EMVI, in our study, EMVI-negative patients presented LC, DFS, and OS values of 100%, 78%, and 84% at 5 years, compared to EMVI-positive patients who showed LC, DFS, and OS values of 80%, 73%, and 64% at 5 years. Also in our experience, statistical significance was evident in LC curves with *p* = 0.01.

### 5.4. Lateral Lymph Nodes (LLN)

Lymph node spread is considered an important factor in tumor local recurrence and represents a primary indication for neoadjuvant therapy. Monocentric studies in 2015 [45] and 2017 [46] demonstrated that CRT with TME is not sufficient to eradicate lateral lymph node disease, resulting in 5-year lateral local recurrence rates of 30% to 40%. Furthermore, some Japanese centers combining CRT with TME and lateral compartment dissection show excellent DFS rates, suggesting that patients with lateral nodal disease can be cured [30]. In the first publication of the Lateral Node Study Consortium with a total of 1216 patients, it was demonstrated that pretreatment LLN sizes of 7 mm or greater resulted in a greatly increased LLR incidence of 19.5%, despite CRT with TME [31].

Therefore, lateral lymph node involvement is receiving increasing interest as a predictive factor. A recent 2024 systematic review analyzed nine studies, totaling 3180 patients, revealing a significant association between LLN presence and increased local recurrence risk (HR: 4.11). It also observed a higher risk for both DFS (HR 1.70) and OS (HR: 1.76) [47]. Ogura et al. observed that, in the 741 patients included in their study, a lateral lymph node size of 7 mm or greater on MRI resulted in a 5-year LC rate of 82%. Lymph nodes positive on pre-CRT MRI and >4 mm on restaging MRI resulted in a 5-year LC rate of 48% [48]. The importance of LLN as a predictive factor for LC is reiterated by two further recent studies: in 2024, the Italian study conducted by Achilli et al. recorded an LC rate in LLN-positive patients of 91% compared to 96% in LLN-negative patients [49]; in 2022, Schaap et al. observed a higher local recurrence rate in the group with LLN compared to the group without LLN (9.8% vs. 2.5%; *p* = 0.056) and also recorded in patients without LLN a significantly better OS than that of patients with LLN detected via MRI (*p* = 0.021) [50].

Recent radiomic models for predicting lateral pelvic lymph node metastasis have demonstrated superior performance compared to conventional size-based criteria, with AUCs reaching 0.819–0.829, and have shown potential for improving clinical decision-making regarding lateral lymph node dissection, as shown in the multicenter retrospective study by Yoo et al. 2025 [32] and in the retrospective study by Zhao et al., 2024 [33].

In our experience, the analysis conducted between LLN+ pre-CRT vs. LLN- pre-CRT groups showed 5-year LC and DFS of 77% and 43% versus 87% and 78%, respectively. The results, although not statistically significant, appear promising with *p* values = 0.10 and 0.20, respectively. Regarding post-treatment analysis, in the group of patients with persistence of LLN, we recorded a statistically significant difference in LC with *p* value = 0.04 and with 5-year rates of 58% for post-CRT LLN+ versus 87% for post-CRT LLN-.

## 6. Summary of Radiomic Features and Clinical Outcomes

The data observed in our study confirmed that radiomic features may serve as indicators of clinical outcomes, showing strong agreement with the literature. The two marked reference studies reported slightly superior performance, possibly due to their selection of a more clinically favorable patient cohort (Table 5).

## 7. The Critical Role of Conventional Features as Clinical Benchmarks

Radiomics can extract quantitative features that reflect tumor heterogeneity and mine data from medical images, going beyond what conventional imaging examinations can provide [51]. However, the journey from conventional morphological assessment to fully integrated AI-driven clinical decision-making requires understanding the strengths and limitations of both approaches. Our study demonstrates that guideline-based morphological features (TEMP > 5 mm, CRM < 1 mm, EMVI presence, pathological LLN) provide robust prognostic stratification using assessment methods already integrated into clinical practice—achieving statistically significant outcome predictions without requiring AI infrastructure.

These findings establish an important clinical benchmark against which AI-based radiomic models can be compared and validated. Recent AI studies in rectal cancer have shown impressive performance metrics: machine learning-based multiparametric MRI radiomics nomograms for perineural invasion prediction achieved AUCs of 0.945 in training sets and 0.846 in validation sets [52], while deep learning algorithms combining radiomics and pathomics for microsatellite instability prediction demonstrated high predictive accuracy with AUCs reaching 0.869–0.923 [53]. However, the clinical utility of these sophisticated models must ultimately be judged against the prognostic value already provided by conventional, accessible morphological assessment.

## 8. Study Limitations

This study has several limitations that should be acknowledged. First, the retrospective single-center design may limit the generalizability of our findings and introduce selection bias. The lack of external validation in an independent cohort prevents definitive conclusions about the reproducibility of our results across different institutions and patient populations.

Second, MRI examinations were not available for review in 41 patients (31%), with 12 not performed due to patient-related issues (pacemaker, metallic prostheses, claustrophobia, or refusal). This missing data may have affected the statistical power of our analyses and potentially introduced bias.

Third, MRI protocols and scanners may have varied over the 10-year study period (2014–2024), potentially introducing technical heterogeneity in image acquisition that could affect morphological feature assessment consistency. Standardization of MRI sequences and parameters is crucial for reliable radiomic analysis. However, this study focuses on morphological features rather than radiomics. Therefore, pixel-level signal harmonization (often required for texture analysis) was not applicable. The consistency of assessment was instead maintained through the use of standardized reporting criteria [12,13,16,28,29].

Fourth, the relatively small sample size for certain subgroup analyses, particularly for lateral lymph node involvement (n = 25 pre-CRT LLN+), may have limited statistical power to detect significant differences and increased the risk of type II errors.

Fifth, the absence of formal inter-observer agreement analysis (ICC or kappa statistics) for the MRI assessments. The collaborative consensus approach, while clinically pragmatic, prevents quantification of measurement reproducibility and potential measurement error. The mixed experience levels of the assessment team, though supervised by senior clinicians, may have introduced variability in feature assessment.

Despite these limitations, our study provides valuable real-world data on MRI-based morphological features as predictive biomarkers in LARC patients treated with dose-intensified neoadjuvant chemoradiotherapy, confirming findings from larger international studies and supporting the clinical utility of these parameters in treatment planning and risk stratification.

## 9. Conclusions

The aims of our experience were to analyze possible radiological factors having predictive value in this patient setting.

Our data appear to be in complete agreement with the evidence in the literature showing mainly a significant impact of treatment on long-term outcomes with very low acute and late toxicities. From our analyses, it emerged that TEMP > 5 mm, circumferential resection margin (CRM) < 1 mm, presence of EMVI, and LLNs were correlated with both lower LC and lower OS and DFS. Better understanding and knowledge of these characteristics, which are already present in the patient’s diagnostic pathway and are easy to perform and interpret, will allow greater personalization of therapy, which can thus be “tailored” to the patient.

Looking forward, the evolution of rectal cancer imaging will likely involve integration of conventional morphological assessment with emerging AI-driven computational radiomics and molecular biomarkers. Such integrated approaches could combine the interpretability and clinical validation of conventional features with the high-dimensional pattern recognition capabilities of machine learning algorithms, potentially providing complementary prognostic information and further optimizing patient stratification.

Future prospective studies and validation in multicenter cohorts are needed to establish standardized protocols for radiomic feature assessment and integration into clinical guidelines.

## Figures and Tables

**Table 2:** Acute toxicity.

Acute Toxicity	G1 (n)	G2 (n)	G3 (n)	G4 (n)
GI	65	28	0	0
GU	37	6	0	0
Skin	17	3	2	0
Hematological	8	3	0	0

Abbreviations: GI: Gastro-intestinal; GU: Genito-urinary.

**Table 3:** Late toxicity.

Late Toxicity	G1 (n)	G2 (n)	G3 (n)	G4 (n)
GI	8	1	1	4
GU	10	1	0	0
Skin	0	0	0	0
Hematological	0	0	0	0

Abbreviations: GI: Gastro-intestinal; GU: Genito-urinary.

**Table 4:** MRI Radiomic Features.

MRI Radiomic Features		Value (n)
Pre-CRT TEMP	Median	6.5 mm
Pre-CRT CRM	Median	3.5 mm
Post-CRT TEMP	Median	2.9 mm
Post-CRT CRM	Median	4.7 mm
Pre-CRT EMVI	Present	62
	Absent	40
Post-CRT EMVI	Present	38
	Absent	64
Pre-CRT LLN	<7 mm	77
	>7 mm	25
Post-CRT LLN	<4 mm	91
	>4 mm	11

Abbreviations: CRT: Chemo-radiation therapy; TEMP: Tumor Extension Beyond Muscularis Propria; CRM: Circumferential Resection Margin; EMVI: Extramural Venous Invasion; LLN: Lateral Lymph Nodes.

**Table 5:** Summary and comparisons about the analyzed MRI features among the selected studies and our experience. * = the results of these two studies are referred only to selected LLN patients with a better clinical presentation. Abbreviations: TEMP: Tumor Extension Beyond Muscularis Propria; CRM: Circumferential Resection Margin; EMVI: Extramural Venous Invasion; LLN: Lateral Lymph Nodes.

Study Authors	TEMP > 5 mm		CRM < 1 mm		EMVI+		LLN+	
	5-yr OS	5-yr DFS	5-yr OS	5-yr DFS	3-yr OS	3-yr DFS	3-yr LC	3-yr OS
Katsumata et al. [36]	53%	60%						
Shin et al. [37]	53%	55%						
Merkel et al. [34]	54%	74%						
MERCURY-Trial [16]			68%	85%				
Patel et al. [41]			46%	51%		44%		
Sun et al. [42]			na	58%	57%	58%		
Wolberick et al. [40]			40–70%	62–58%				
Chand et al. [21]						59%		
Lee et al. [47]						44%		
Ogura et al. [48]							82%	na
Achilli et al. [49]							91% *	na
Schaap et al. [50]							90% *	55% *
Our experience	66%	68%	67%	74%	64%	73%	78%	45%

## Data Availability

The dataset generated and analyzed for the current study is not publicly available but is available from the corresponding author upon request.
